# Characterizing Microbial Diversity and the Potential for Metabolic Function at −15 °C in the Basal Ice of Taylor Glacier, Antarctica

**DOI:** 10.3390/biology2031034

**Published:** 2013-07-26

**Authors:** Shawn M. Doyle, Scott N. Montross, Mark L. Skidmore, Brent C. Christner

**Affiliations:** 1 Department of Biological Sciences, Louisiana State University, Baton Rouge, LA 70803, USA; E-Mail: sdoyle2@gmail.com; 2 Department of Earth Sciences, Montana State University, Bozeman, MT 59717, USA; E-Mails: smontross@montana.edu (S.N.M.); skidmore@montana.edu (M.L.S.)

**Keywords:** Antarctica, basal ice, subzero metabolism, microbial survival

## Abstract

Measurement of gases entrapped in clean ice from basal portions of the Taylor Glacier, Antarctica, revealed that CO_2_ ranged from 229 to 328 ppmv and O_2_ was near 20% of the gas volume. In contrast, vertically adjacent sections of the sediment laden basal ice contained much higher concentrations of CO_2_ (60,000 to 325,000 ppmv), whereas O_2_ represented 4 to 18% of the total gas volume. The deviation in gas composition from atmospheric values occurred concurrently with increased microbial cell concentrations in the basal ice profile, suggesting that *in situ* microbial processes (*i.e.*, aerobic respiration) may have altered the entrapped gas composition. Molecular characterization of 16S rRNA genes amplified from samples of the basal ice indicated a low diversity of bacteria, and most of the sequences characterized (87%) were affiliated with the phylum, Firmicutes. The most abundant phylotypes in libraries from ice horizons with elevated CO_2_ and depleted O_2_ concentrations were related to the genus *Paenisporosarcina*, and 28 isolates from this genus were obtained by enrichment culturing. Metabolic experiments with *Paenisporosarcina* sp. TG14 revealed its capacity to conduct macromolecular synthesis when frozen in water derived from melted basal ice samples and incubated at −15 °C. The results support the hypothesis that the basal ice of glaciers and ice sheets are cryospheric habitats harboring bacteria with the physiological capacity to remain metabolically active and biogeochemically cycle elements within the subglacial environment.

## 1. Introduction

During freezing, soluble and insoluble impurities (solutes, microbes, particles and gases) are physically excluded from the ice crystal lattice and concentrated into saline veins of liquid water found at the interface between ice crystals [1]. Despite the presence of liquid water, ice veins are environments in which microorganisms must endure physiochemical stresses such as low water activity, low pH, and ice recrystallization, as well as the biochemical challenges associated with low temperatures (e.g., reduced enzymatic activity and decreased membrane fluidity) [2]. There are two mechanisms by which microorganisms can be incorporated in glacial ice: aeolian deposition at the surface and entrainment of sediments in the basal zone [3,4]. Once entrapped in the ice, the long-term survival of a microbial population is constrained by their capability to endure the genetic and cellular damage that would accumulate in the absence of a functional metabolism. Damage to cellular macromolecules can be caused by a variety of physical and chemical mechanisms, including natural background ionizing radiation (e.g., produced from the decay of ^40^K, ^232^Th and ^238^U), L-amino acid racemization and spontaneous hydrolysis or oxidation of DNA [2,5]. As such, metabolically dormant microbial populations that remain frozen for extended periods of time would eventually accumulate a lethal amount of damage [5]. However, microorganisms with the capability to maintain a low level of metabolism requisite for mitigating genetic and cellular damage could theoretically persist, as long as suitable redox couples and nutrients were available to support their metabolic activity. Hence, the discovery of viable microbes persisting in ancient ice and permafrost [6,7,8,9,10,11,12] gives credence to the hypothesis that certain microorganisms are actively maintaining their cellular integrity under these conditions.

Analysis of gases entrapped in ice cores of glacial and basal ice from Antarctica (Siple Dome, Vostok), Greenland (North Greenland Ice Core Project, Greenland Ice Core Project) and South America (Sajama ice cap, Bolivia) have found concentrations of N_2_O, CO_2_ and CH_4_ and stable isotopic compositions of N_2_O and CH_4_ [13,14,15,16,17,18] that do not correspond to atmospheric values. Microbial processes, such as nitrification and methanogenesis, are plausible explanations for the low δ^18^O-N_2_O and δ^13^C-CH_4_ values, respectively [17,19,20]. In support of this, laboratory studies have shown that microorganisms remain metabolically active at subzero temperatures, including respiration at temperatures of −33 °C [21] and −39 °C [22] and macromolecular synthesis at −15 °C [5,23,24]. Nevertheless, there are few data on the nature and constraints of *in situ* microbial activity in natural icy systems, and knowledge of subzero microbial physiology and its role in subglacial biogeochemical cycling is limited. Here, we present results from an investigation of microbial assemblages within basal ice horizons of Taylor Glacier, Antarctica. Basal ice is found in the deepest layers of a glacier and has a chemistry and physical structure that is directly affected by its proximity to the glacier bed [25]. Sedimentary debris becomes entrained in the ice at the basal zone, together with viable microorganisms and substrates suitable as energy and nutrient sources, which may create unique habitats within the ice [12,26,27,28]. The specific aim of this research was to investigate the potential for basal ice to serve as a microbial habitat, with the implication that microorganisms are ultimately responsible for the unusual concentration of gasses (e.g., CO_2_ and O_2_) found entrapped in these icy environments. Our data on active biogeochemical processes in the basal zone of Taylor Glacier is discussed in the broader context of polar ice sheets and potential habitats for life in icy extraterrestrial frozen environments. 

## 2. Methods

### 2.1. Site Information and Field Sampling

**Figure 1:**
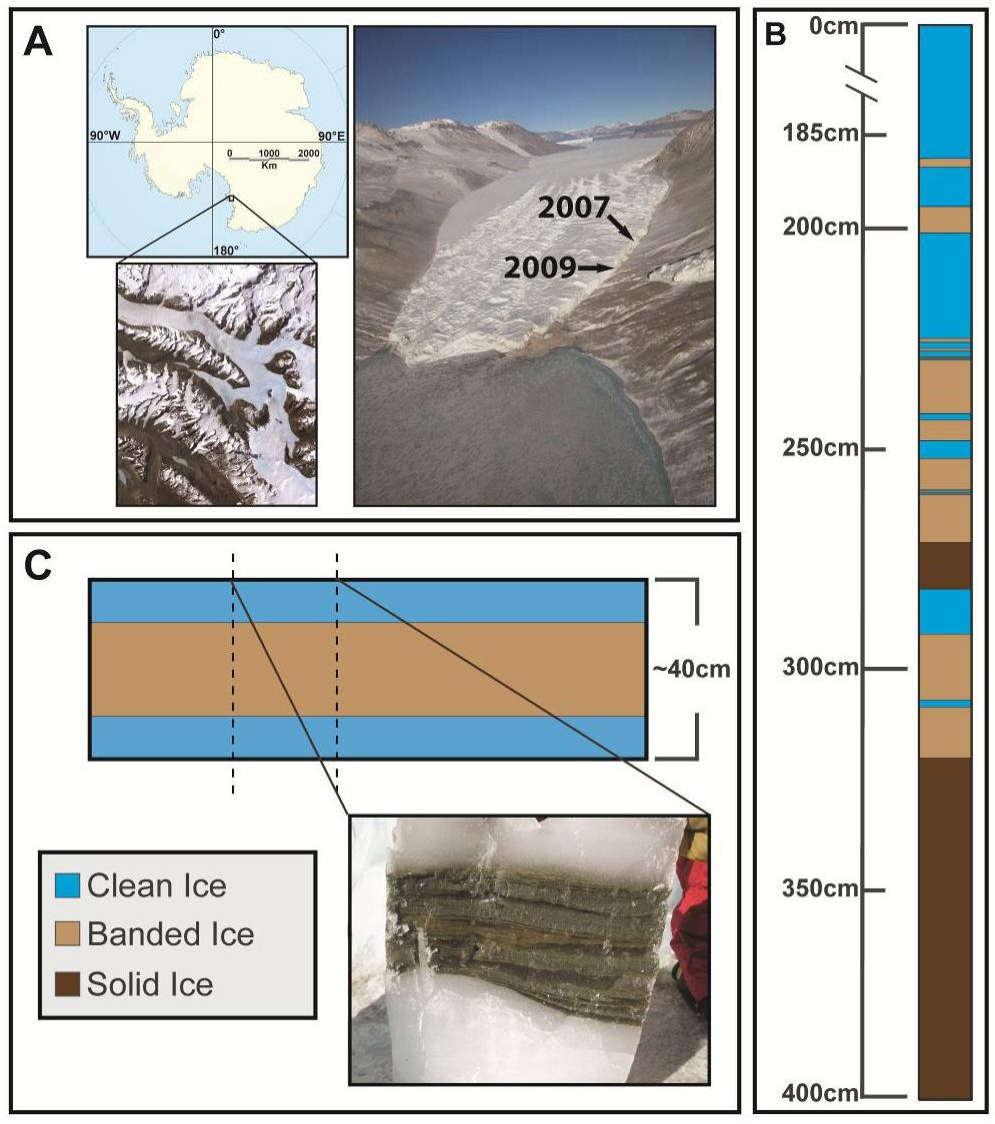
(**A**) Map and aerial photograph of Taylor Glacier, located in the McMurdo Dry Valleys of Victoria Land, Antarctica. The location of the 2007 and 2009 access tunnels are indicated. (**B**) Schematic of the 4 m deep basal ice profile sampled from Taylor Glacier in 2007. The top of the profile (designated 0 cm) was located in debris-poor clean ice, which was underlain by several layers of both debris-rich banded dispersed and laminated solid ice with a thick layer of basal solid ice as the lowermost unit. (**C**) Schematic of the debris-rich banded dispersed basal ice horizon sampled in 2009.

Taylor Glacier is a 54 km outflow glacier of the East Antarctic Ice Sheet and is located at the western end of Taylor Valley in the McMurdo Dry Valleys of Victoria Land, terminating on the western shore of Lake Bonney (Figure 1A). During the austral summers of 2007 and 2009, two tunnels were excavated into the northern margin of Taylor Glacier to directly access a stratigraphic sequence of basal ice that was largely free of folding or distortions found in horizons at the margin. The tunnels were initiated on fresh ice aprons and extended 7–9 m in from the ice margin. In 2007, a vertical shaft (~5 m) was constructed at the end of the tunnel, and a 4 m vertical profile of basal ice was sampled. Three distinct basal ice facies were identified using the nomenclature of Hubbard *et al*. [29]: (i) clean ice, containing <1 g L^−1^ debris; (ii) banded dispersed ice, containing debris up to 38% w/v; and (iii) solid ice, which is heavily debris laden (up to 60% w/v). The 4 m vertical profile collected contained ice from all three facies (Figure 1B), and the top of the sample profile (*i.e.*, access tunnel floor) was designated as the zero depth. Sample ice blocks measuring approximately 20 × 20 × 10 cm were cut using electric chainsaws with carbide tipped chains. During the 2009 season, a new access tunnel was excavated to directly intersect a layer of debris-rich banded dispersed ice (Figure 1C), and 27 large (40 × 30 × 15 cm) blocks of banded ice were collected. Temperature loggers deployed in the basal ice during the 2007 season indicated an ice temperature of −15 °C. All ice samples were shipped frozen to Montana State University and Louisiana State University and stored at −20 °C. Gas measurements for the ice samples are described in Montross [30] and Montross *et al*. [31].

### 2.2. Ice Decontamination and Sampling

The debris-free ice was subsequently cut using a band saw, and samples of the sediment-laden ice were cut using a masonry saw equipped with a diamond blade. The ice samples were handled with sterile stainless steel forceps and decontaminated in a class 100 laminar flow hood housed within a −5 °C freezer. The surface contaminated outer portion of the ice was removed based on a method developed by Christner *et al*. [32] for sampling deep ice cores recovered in boreholes containing hydrocarbon-based drilling fluids. The outermost surface of the ice sample was washed with 0.22 μm filtered 95% ethanol that was equilibrated to −5 °C. Samples were then rinsed with ice-cold 0.22 μm filtered, twice-autoclaved deionized water until an estimated minimum of ten millimeters of the outer sample surface had been removed. Sterile forceps were used to hold the samples during washing and were exchanged frequently to prevent carryover contamination. All samples were weighed before and after decontamination, and the decontamination method reduced the total ice mass of each sample by 15% to 25%. The cleaned samples were placed in sterile containers and melted at 4 °C (typically 16 h to 24 h).

### 2.3. Microbial Cell Density

Sections of both the clean ice and banded dispersed ice from the 2007 sample profile were selected for microscopic cell counts. Within a −5 °C freezer, a profile of the clean ice (depth 60–80 cm) was cut and sampled at a vertical resolution of 5 cm; the banded dispersed ice (depth 220–240 cm) was sampled at a vertical resolution of 2.5 cm. The ice samples were subsequently decontaminated and melted as described above (Section 2.2).

For enumeration, microbial cells attached to sediment particles were liberated from the solid phase with a modification of the method described by Trevors and Cook [33]. Nine milliliters of the sediment-melt water slurry was amended with 1 mL of a 1% (w/v) solution of Na_4_P_2_O_7_ (pH 7.0), shaken at 200 rpm for 1 h at 4 °C and allowed to settle for 30 min. The supernatant was collected, and the cells within were fixed with sodium borate-buffered formalin (5% final concentration), stained with 2× SYBR Gold (Invitrogen) and filtered onto black polycarbonate 0.22 μm pore filters (GE Water & Process Technologies). Identical samples that did not contain the formalin fixative were also prepared, stained with Baclight (Invitrogen) and filtered within 6 h after melting. The filters were mounted on glass slides with a glass coverslip using two drops of antifade solution and stored in the dark at 4 °C until counted. The antifade solution consisted of 90 mM *p*-phenylenediamine and 45% glycerol in phosphate buffered saline and was filtered through a 0.45 μm filter. Fifty random fields (field of view: 41,500 μm^2^) were counted using an Olympus BX51 epifluorescence microscope and a FITC filter cube (excitation from 455 to 500 nm and emission from 510 to 560 nm). Cell density estimates were calculated based on the average number of cells per field and normalized per gram of ice. The sediment content for each sample was determined by measuring the dry weight of sediment per gram of basal ice. 

### 2.4. Enrichment and Isolate Culturing

Meltwater from the debris-rich banded dispersed ice in the 2007 sample profile (depth 195 cm to 200 cm; Figure 1) was vortexed for 1 min, and 100 μL of the slurry was spread plated on R2A (Difco), 10% R2A, 1% R2A, marine agar 2216 (Difco) and M9 minimal salts media (supplemented 20 mM glucose, acetate or pyruvate) in triplicate. The plates were incubated at 4, 10, 22 and 37 °C in the dark and examined daily for 60, 30, 15 and 7 days, respectively. Blank media controls were prepared and incubated in parallel with inoculated samples. Additional isolates from ice samples collected from a tunnel constructed at Taylor Glacier in 1999 were made available for this investigation; details of the tunnel location and physical and chemical properties of the ice are described in Samyn *et al.* [34]. Growth at 5, 15 and 22 °C was measured via optical density (620 nm) in marine broth 2216 (Difco) to determine the approximate optimal growth temperature of each isolate. Pasteurization of the melted ice was performed by heating at 80 °C for 10 min, followed by spread plating 100 µL of the sample on marine agar 2216 (Difco) in triplicate. The cultures were incubated aerobically at 22 °C, and the number of colony-forming units (CFU) was quantified and compared to control samples. Marine agar 2216 (Difco) consistently yielded the highest CFU mL^−1^ from samples, and therefore, was used for this assay. 

Salt tolerance of select isolates was examined by culturing in marine broth 2216 (Difco) supplemented with up to 10% (w/v) of NaCl (intervals of 2% NaCl). Optical density (OD_620__nm_) of the cultures was monitored at 10 °C over two weeks using a NanoDrop spectrophotometer.

### 2.5. Molecular Analysis of Bacterial 16S rRNA Genes

**Figure 2:**
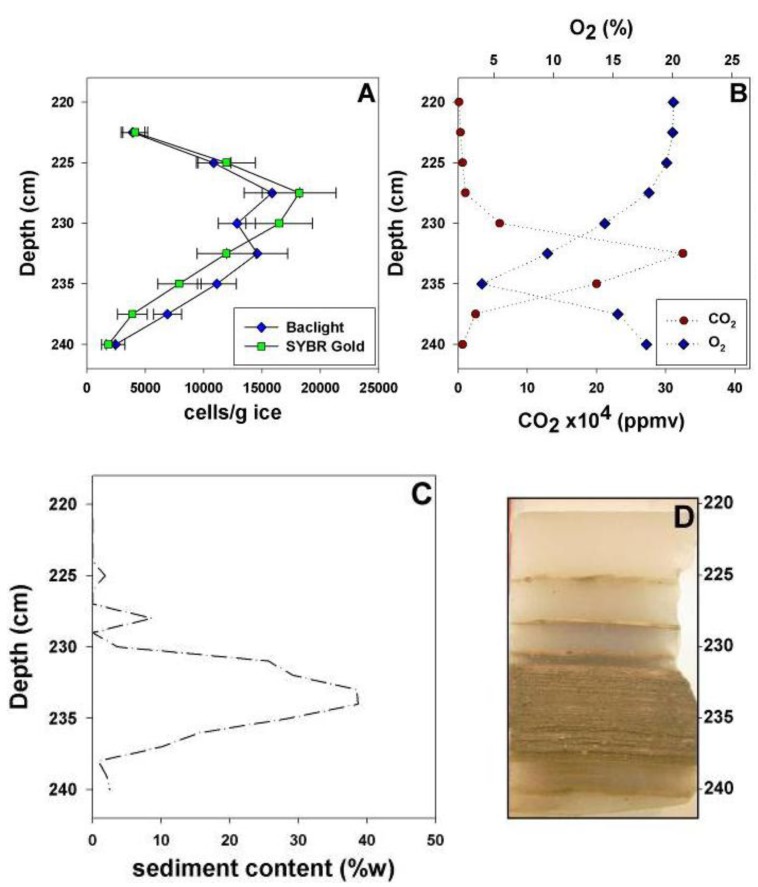
Analysis of the microbial cell density (**A**), concentrations of O_2_ and CO_2_ (**B**) and sediment content (**C**) throughout a vertical profile of banded dispersed basal ice (**D**). Error bars represent the standard error of the direct cell counts.

Genomic DNA was extracted from the banded dispersed basal ice facies recovered in 2007 and 2009. For the sample from the 2007 profile, an entire sample block (20 × 20 × 10 cm, profile depth 220–240 cm (Figure 2)) was decontaminated and melted at 4 °C. The resulting meltwater was vigorously shaken (300 rpm) to achieve a homogenous sediment-meltwater slurry, 15 mL of which was centrifuged (4,500 × g; 10 min; 4 °C), and total DNA was extracted from 0.5 g of the sediment pellet using a MoBio PowerSoil DNA extraction kit, as per the manufacturer’s instructions. For samples collected in 2009, ~132 kg of basal ice was selected for filter concentration prior to shipment back to the United States from Antarctica. After decontamination, the ice was placed at 4 °C in sterilized polypropylene containers and allowed to melt. Complete melting of this basal ice took place over a period of seven days, wherein the meltwater was concentrated onto filters. In order to remove larger sediment particles, the sediment-meltwater slurry was filtered consecutively through a series of five sterilized nylon monofilament filters of decreasing pore size (100, 75, 50, 25 and 10 μm) and, then, centrifuged at 700 × g (10 min; 4 °C) [35]. The supernatant (~90 L) was filtered at 4 °C under a 20 cm Hg vacuum onto eleven 90 mm, 0.22 μm Supor-200 filters (Pall Corporation). The filters were frozen at −80 °C and shipped to Louisiana State University for storage and analysis. DNA was extracted from one of the 90 mm filters through which 5.1 L of meltwater had been filtered using a MoBio PowerMax soil DNA extraction kit. 

A portion of the 16S rRNA gene was amplified from the extracted genomic DNA samples using the primers, 27F (5'-AGAGTTTGATCCTGGCTCAG-3') and 1492R (5'-GGTTACCTTGTTACGACTT-3') [36]. For the 2009 gDNA extracted from banded dispersed ice, the 50 μL PCR reaction contained 1.0 unit of *Taq* DNA polymerase (5PRIME), 1× Master*Taq* buffer, 1× *Taq*Master PCR enhancer, 1.5 mM Mg(C_2_H_3_O_2_)_2_, 15 pmol of each primer, 200 μM deoxynucleotide triphosphates (dNTPs), and ~100 pg of template DNA. Thirty cycles of PCR were done with a 45 s denaturation step at 94 °C, 60 s annealing step at 50.8 °C and extension at 72 °C for 60 s, followed by a final extension at 72 °C for 10 min. The 50 μL PCR reaction for the 2007 gDNA sample contained 5.0 units of AmpliTaq Gold DNA polymerase, LD (Invitrogen), 1× PCR Gold buffer, 3.5 mM Mg(C_2_H_3_O_2_)_2_, 30 pmol of each primer, 200 μM dNTPs and ~100 pg of template DNA. Forty-three cycles of time-release PCR were done with a 45 s denaturation step at 94 °C, 60 s annealing step at 50.8 °C and extension at 72 °C for 60 s, followed by a final extension at 72 °C for 10 min.

The PCR products obtained were examined by agarose gel electrophoresis, purified by ethanol precipitation and ligated into the pGEM T-Easy plasmid (Promega). Alpha complementation was used to identify clones containing inserts, and inserts of the predicted size were confirmed by PCR with primers that annealed to the flanking SP6 and T7 regions of the vector. Each clone was cultured in Luria Bertani medium amended with 100 μg mL^−1^ of ampicillin, and plasmid DNA was purified using the Qiagen MiniPrep kit, quantified on a Nanospec spectrophotometer and sequenced using BigDye Terminator (v. 3.1; Invitrogen) on an ABI 3130XL Genetic Analyzer (Applied Biosystems). The forward and reverse sequencing reads were manually trimmed to remove flanking vector and primer sequences and aligned with BioEdit software. The compiled sequences were aligned with SINA (v. 1.2.11) [37] using the SILVA reference database (release 113) [38]. Phylogenetic classification, diversity estimation and rarefaction were performed in MOTHUR [39]. For the diversity estimation, operational taxonomic units (OTUs) were identified at a genetic distance of 3%. The UCHIME algorithm [40] was used in MOTHUR to identify potential chimeric sequences, which were discarded from the analysis. Maximum likelihood phylogenetic trees were constructed in MEGA5 [41]. 

A total of 25 isolates were chosen for 16S rRNA gene sequencing based on differences in colony morphology, pigmentation, growth temperature and media of isolation (Table 1). Genomic DNA was extracted from each isolate using the UltraClean Microbial DNA isolation kit (MoBio Laboratories). Bacterial 16S rRNA genes were amplified from the genomic DNA by PCR using the primers, 27F and 1492R [36]. The 50 μL PCR reactions contained 1.0 units of MasterTaq DNA polymerase (5 PRIME), 1× Taq buffer, 1× TaqMaster PCR enhancer, 1.5 mM Mg (C_2_H_3_O_2_)_2_, 15 pmol of each primer, 200 μM dNTPs and ~300 ng of template DNA. Thirty cycles of amplification were performed with denaturation for 60 s at 96 °C, annealing at 50.8 °C for 1 min, extension at 72 °C for 2 min and a final extension for 10 min at 72 °C. PCR products of the expected length (≈1,500 bp) were purified by ethanol precipitation and sequenced as described above. Taxonomic assignments were performed using EzTaxon-E [42].

The DNA sequences obtained in this study were deposited in the GenBank database under accession numbers, KC777190 to KC777289.

**Table 1:** Phenotypic description of basal ice isolates and their phylogenetic relationships to cultured bacteria. N.D. = not determined.

Isolate	Closest Relative	% Identity	Sequence Length (bp)	Isolation Media	Isolation temp	Optimal temp (±5 °C)	Halotolerance (% NaCl)	Description
TG14	*Paenisporosarcina antarctica* N-05	99	1,379	R2A	10 °C	15 °C	~7.9% (w/v)	bright yellow, circular, flat
TG24	*Paenisporosarcina antarctica* N-05	99	1,363	marine	10 °C	15 °C	~9.9% (w/v)	tan, circular, convex
TG27	*Paenisporosarcina antarctica* N-05	99	1,366	M9 glucose	4 °C	15 °C	~7.9% (w/v)	off-white, shiny, convex
TG29	*Paenisporosarcina antarctica* N-05	99	1,364	M9 glucose	4 °C	22 °C	N.D.	yellow, shiny, convex
TG30	*Paenisporosarcina antarctica* N-05	99	1,369	M9 pyruvate	4 °C	22 °C	N.D.	white, shiny, convex
TG32	*Paenisporosarcina antarctica* N-05	99	1,364	10% R2A	4 °C	22 °C	N.D.	off-white, circular, shiny
TG25	*Paenisporosarcina antarctica* N-05	99	1,363	marine	10 °C	15 °C	N.D.	cream-yellow, shiny, convex
TG34	*Paenisporosarcina antarctica* N-05	100	1,368	1% R2A	4 °C	22 °C	N.D.	off-white, circular
TG21	*Paenisporosarcina macmurdoensis* CMS21w	99	1,367	marine	22 °C	22 °C	~7.9% (w/v)	bright yellow, rough
TG3	*Paenisporosarcina macmurdoensis* CMS21w	99	1,375	R2A	22 °C	22 °C	~5.9% (w/v)	off-white center, mucoid
TG6	*Paenisporosarcina macmurdoensis* CMS21w	99	1,382	1% R2A	22 °C	22 °C	~5.9% (w/v)	white-yellow, mucoid
TG7	*Paenisporosarcina macmurdoensis* CMS21w	99	1,387	1% R2A	22 °C	22 °C	N.D.	off-white, mucoid
TG18	*Paenisporosarcina macmurdoensis* CMS21w	99	1,349	R2A	22 °C	22 °C	N.D.	off-white, mucoid
TG11	*Paenisporosarcina macmurdoensis* CMS21w	99	1,373	R2A	22 °C	15 °C	N.D.	cream yellow, circular
TG2	*Paenisporosarcina macmurdoensis* CMS21w	99	1,382	R2A	22 °C	22 °C	N.D.	cream yellow, mucoid
TG15	*Paenisporosarcina macmurdoensis* CMS21w	99	1,376	R2A	10 °C	22 °C	N.D.	yellow, mucoid
TG17	*Paenisporosarcina macmurdoensis* CMS21w	99	1,377	R2A	10 °C	22 °C	N.D.	white-yellow, convex, mucoid
TG26	*Paenisporosarcina macmurdoensis* CMS21w	99	1,369	marine	10 °C	22 °C	N.D.	off-white, mucoid
TG19	*Paenisporosarcina indica*	99	1,369	marine	22 °C	22 °C	~7.9% (w/v)	dark brown, convex
TG9	*Paenisporosarcina indica*	99	1,372	R2A	22 °C	22 °C	~7.9% (w/v)	dull yellow, translucent, flat, mucoid
TG20	*Paenisporosarcina indica*	99	1,386	marine	22 °C	22 °C	N.D.	cream-yellow, convex
TG39	*Paenisporosarcina indica*	99	1,370	marine	4 °C	22 °C	N.D.	white, shiny, convex
TG10	*Paenisporosarcina quisquiliarum* SK 55	99	1,383	R2A	22 °C	22 °C	~5.9% (w/v)	off-white, shiny, convex
TG8	*Bacillus humi* LMG18435	97	1,367	1% R2A	22 °C	22 °C	~5.9% (w/v)	tan with brown center, circular
TG4	*Paraliobacillus quinghaiensis* YIMC158	99	1,418	R2A	22 °C	15 °C	~11.9% (w/v)	yellow, convex, rough

### 2.6. DNA and Protein Synthesis of Isolated Bacteria at −15 °C

Measurement of DNA and protein synthesis by cells frozen at −15 °C was carried out based on the procedure described by Christner [23]. Cultures (50 mL) of *Paenisporosarcina* sp. TG14 were grown aerobically (200 rpm) at 15 °C in marine broth 2216 (Difco). Cells were harvested from mid-exponential phase cultures by centrifugation (10 min, 4,500 × g). The harvested cells were then suspended in 50 mL of Taylor Glacier melt water, centrifuged, and suspended in 50 mL melt water at a concentration of 3.1 × 10^6^ CFU mL^−1^. Experiments were conducted using melt water from both debris-poor clean ice and debris-rich banded dispersed ice. The samples used for these experiments were taken adjacent to the main vertical sampling profile at sample depths of approximately ~100 cm depth for the clean ice and ~300 cm for the banded dispersed ice. Due to their opaque nature, sediment particles readily quench luminescence during liquid scintillation counting, and thus decrease measurement reliability. To mitigate this effect, coarse particles were allowed to settle from the samples for 18 h prior to harvesting melt water for these experiments. Aliquots (500 μL) of the cell suspension were amended with 1.7 μCi mL^−1^ of [^3^H]-leucine (L-leucine [4,5-^3^H], 84 Ci mmol^−1^ in ethanol:water 2:98; MP Biomedical) or 1.3 μCi mL^−1^ [^3^H]-thymidine (thymidine [Methyl-^3^H], 64 Ci mmol^−1^ in sterile water; MP Biomedical) to achieve a final concentration of 20 nM. Killed controls were amended with ice-cold 50% trichloroacetic acid (TCA) to a final concentration of 7% (w/v) 30 min prior to the addition of [^3^H]-leucine or [^3^H]-thymidine. All solutions were maintained on ice, and the samples were placed in a −80 °C freezer within 30s after the addition of either [^3^H]-leucine or [^3^H]-thymidine. After overnight (16 h) incubation at −80 °C, samples were transferred to a −15 °C thermally stable freezer (Revco ULT350-3-A32), which was designated as time zero. A HOBO U12 (Onset) data logger was used to log the temperature in the freezer every 10 min. Over the entire experimental time course, the mean temperature was −15.0 ± 0.5 °C. At each experimental time-point, frozen samples were removed from the freezer, immediately overlain with 100 μL of ice-cold 50% TCA, briefly centrifuged (10 s), and incubated at 4 °C to allow melting (final TCA concentration was 7%). After at least 30 min, the acid-insoluble macromolecules were pelleted by centrifugation at 17,000 × g for 15 min; the pellet was rinsed with 1 mL of ice-cold 5% TCA and centrifuged at 17,000 × g for 5 min. The residual was rinsed with 1 mL of ice-cold 70% ethanol, centrifuged at 17,000 × g for 5 min and the supernatant removed. The rinsed pellet was suspended in 1 mL of Cytoscint scintillation cocktail (Fisher, cat. no. BP458-4), and the radioactivity present was quantified using liquid scintillation spectrometry (Beckman LS6000IC scintillation counter). The number of disintegrations per minute (DPM) was calculated by determining counting efficiency using acetone-quenched standards of [^3^H] toluene (American Radiolabeled Chemicals, cat# ARC182) in the Cytoscint cocktail. Incorporation rates per CFU were calculated over time and converted to molecules of substrate incorporated, per CFU, per day (molecules CFU^−1^ day^−1^). Rate measurements were converted to grams of substrate carbon incorporated, per gram of cell carbon, per day (gC gC^−1^ day^−1^) based on 65 fg C cell^−1^ [5]. A rectangular hyperbole (*i.e.*, one site saturation curve) was used as a best fit to model the data. 

## 3. Results

### 3.1. Cell Concentration and Viability within Horizons of the Basal Ice

Direct counting using epifluorescent microscopy was used to quantify cell density in sampled horizons of the basal ice. The debris-poor clean ice horizons (2007 profile: 60 cm to 80 cm; selected as a representative piece of the clean ice) contained the lowest total cell concentrations of the entire basal ice profile: SYBR Gold and Baclight staining revealed counts ranging from 2.6 ± 0.2 to 4.9 ± 0.4 × 10^2^ cells g^−1^ ice (n = 4). A two-way ANOVA was conducted to test for differences in cell concentrations in the clean ice, and the data did not differ significantly between sampling depths within the 60–80 cm block [*F*(3, 196) = 1.23, *p* = 0.30]. In the debris-rich banded dispersed ice (profile depth 220 cm to 240 cm), total cell concentrations were approximately one to two orders of magnitude higher than the clean ice. SYBR Gold counts ranged from 1.8 ± 0.1 × 10^3^ cells g^−1^ to 1.8 ± 0.4 × 10^4^ cells g^−1^, while Baclight counts ranged from 2.4 ± 0.1 × 10^3^ cells g^−1^ to 1.6 ± 0.3 × 10^4^ cells g^−1^ (Figure 2A); cell abundance estimates by the two methods were not significantly different (α = 0.05). Cell concentration was positively correlated with sediment content [*r*(14) = 0.60, *p* < 0.05] and the concentration of CO_2_ [*r*(14) = 0.35, *p* < 0.10; Figure 2]. In horizons with increased sediment content (profile depth 230–237.5 cm; Figure 2C), sediment content was correlated with CO_2_ [*r*(6) = 0.72, *p* < 0.025] and O_2_ [*r*(6) = −0.79, *p* < 0.01], whose concentrations were elevated and depleted, respectively, relative to atmospheric values [30,31]. Based on Baclight staining, cell viability in the banded dispersed ice was estimated at 73 ± 9%, did not vary with sediment content, and was not significantly different (α = 0.05) from the debris-poor clean ice (78 ± 5%).

### 3.2. Enrichment Culturing from the Basal Ice

Growth was observed on all media inoculated and incubated at 4, 10 and 22 °C, except M9 supplemented with acetate as a carbon source. No growth was observed at 37 °C. Enrichment culturing of heterotrophic bacteria at 22 °C with marine media (Difco 2216) in triplicate yielded an average of 9.7 ± 1.5 × 10^1^ CFU mL^−1^ in the banded dispersed ice melt-water from 195–200 cm, indicating that *ca.* 0.7% of the cells observed via SYBR Gold and Baclight staining were culturable under the conditions used. Pasteurization with duplicate melt-water samples was performed to determine if the cells cultured were in a vegetative state (*cf*., endospores) in the melted ice samples. Pasteurization of the meltwater reduced the recovery of viable cells by 96%. All of the isolates had an optimal growth temperature between 15 °C and 22 °C; moreover, all isolates were capable of growth at 4 °C (the lowest temperature tested). Of the isolates examined for halotolerance, all grew optimally in marine broth and had reduced growth rates when the marine broth was amended with additional NaCl. For the eleven isolates tested, the maximum salt concentration that supported growth was between ~5.9% and ~11.9% (w/v) NaCl (Table 1).

### 3.3. Phylogenetic Analysis of Clone Sequences and Isolates

**Figure 3:**
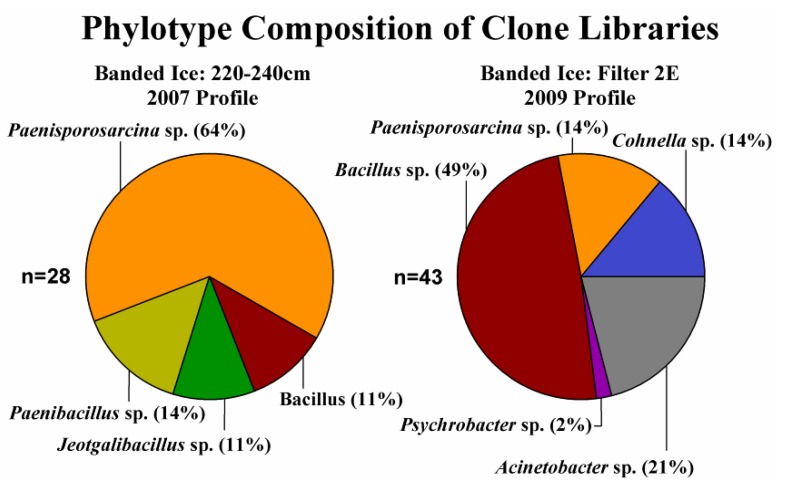
Taxonomic classification of partial 16S rRNA gene sequences amplified and cloned from the Taylor Glacier banded ice. Phylogenetic assignments were performed after 100 iterations using the naive Bayesian classifier [43] in MOTHUR with the SILVA database as a reference.

Forty-three clones were sequenced from the clone library constructed from the 2009 banded dispersed basal ice sample, none of which possessed properties typical of chimeras. The 16S rRNA gene sequence clones in this library were phylogenetically related to the Firmicutes (77%) and the Gammaproteobacteria (Figure 3). The Firmicute-related clones were related to three genera: *Bacillus*, *Paenisporosarcina* and *Cohnella*, with *Bacillus*-related phylotypes being the most abundant (49%) in the library. The Gammaproteobacteria were represented by only two genera, *Acinetobacter* and *Psychrobacter*. For the 2007 banded dispersed ice gDNA, attempts at amplifying the 16S rRNA gene with MasterTaq as described above were not successful. However, following the manufacturer’s recommendations for amplification of targets with low template concentrations, successful amplification of the 16S rRNA gene sequence was achieved using the AmpliTaq Gold DNA polymerase. Forty-one clones were sequenced from the 16S rRNA gene clone library constructed using DNA obtained from the 2007 banded dispersed basal ice sample (profile depth 220–240 cm), thirteen of which were discarded as potential chimeras or PCR artifacts. All the clones sequenced were related to members of four genera within the Firmicutes: *Paenibacillus*, *Paenisporosarcina*, *Bacillus* and *Jeotgalibacillus*. Sequences related to the *Paenisporosarcina* were the most commonly observed phylotype in the clone library, representing 65% of all the sequences characterized. Four clone sequences could not be classified beyond the phylum level and were, thus, listed as unclassified Firmicutes. Shannon diversity indices for both clone libraries were similar: 2007 H’ = 1.81 ± 0.42; 2009 H’ = 1.78 ± 0.22.

**Figure 4:**
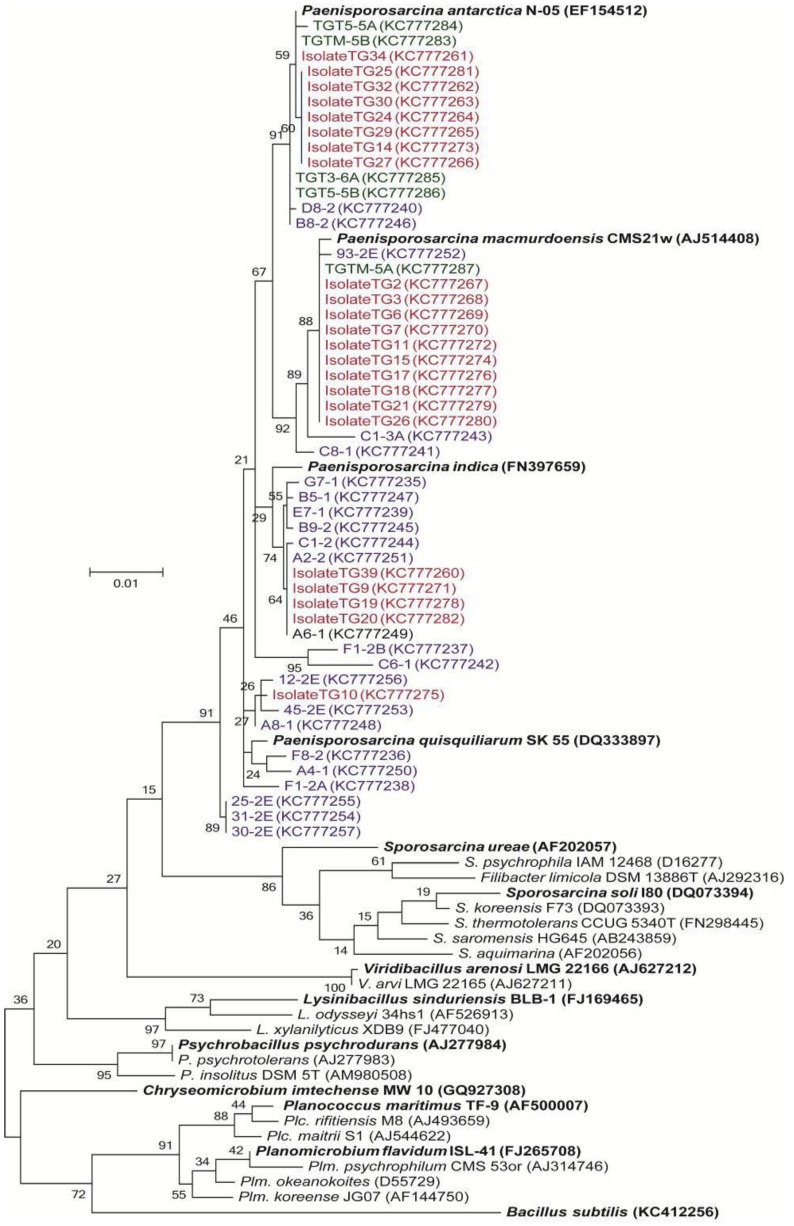
Phylogenetic tree of *Paenisporosarcina*-related clone and isolate 16S rRNA gene sequences using a Maximum Likelihood method based on the Jukes-Cantor model. The partial 16S rRNA gene sequences, corresponding to nucleotides 108–1407 (*E. coli* numbering), were aligned to the SILVA reference database (v.113). After filtering gaps, the final alignment was based on 1,288 nucleotides. Isolate sequences are shown in red and cloned sequences are shown in blue. Isolates recovered from the 1999 samples are shown in green. The bootstrap consensus tree inferred from 1,000 replicates is taken to represent the evolutionary history of the taxa analyzed [44]. Branches corresponding to partitions reproduced in less than 50% bootstrap replicates are collapsed. The percentage of replicate trees in which the associated taxa clustered together in the bootstrap test is shown next to the branches. The scale bar represents the number of changes per nucleotide position.

The cultured isolates belonged to three genera in the bacterial phylum, Firmicutes: *Paenisporosarcina*, *Bacillus* and *Paraliobacillus* (Table 1). Members of the *Paenisporosarcina* were the most abundant taxa cultured, representing 28 out of the 30 unique isolates characterized. The 16S rRNA gene sequences from the *Paenisporosarcina-*related isolates were similar (≥97% identity) and clustered with sequences obtained in the clone libraries (Figure 4). Some of the clone sequences were 100% identical to those obtained from cultured isolates. Five of the isolates (shown in green in Figure 4) were recovered from Taylor Glacier basal ice samples obtained from the 1999 tunnel site [34]. 

### 3.4. Incorporation of Macromolecular Precursors in Melt water at −15 °C

**Figure 5:**
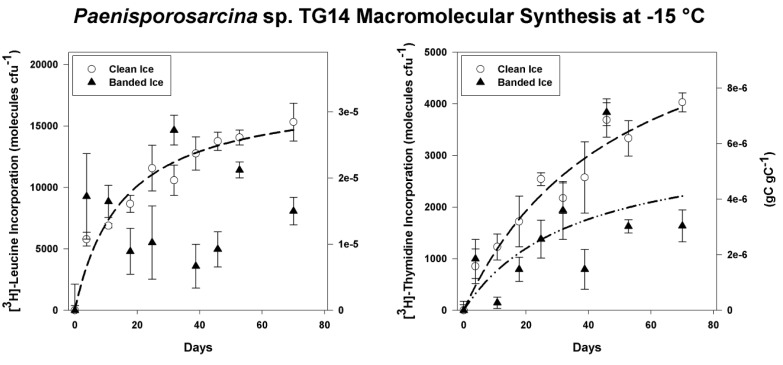
[^3^H]-Leucine and [^3^H]-thymidine incorporation into trichloroacetic acid (TCA) precipitable material at −15 °C by frozen cell suspensions of *Paenisporosarcina* sp. TG14 in clean ice melt water (white circles) and banded dispersed ice melt water (black triangles). The initial cell concentration of the TG14 cell suspensions was 3.1 × 10^6^ colony-forming units (CFU) mL^−1^. Error bars are the standard error of triplicate samples. Incorporation is reported as molecules of substrate incorporated per CFU on the left axis and grams of substrate carbon incorporated per gram of cell carbon on the right-hand axis. Where significant (*p* < 0.05), the best-fit regression curves (hyperbolic) are plotted as dashed lines.

Isolate *Paenisporosarcina* sp. TG14 had the fastest growth rate of the three isolates tested (TG14, TG19 and TG21) at 15 °C (3.0 h generation^−1^) and was selected for a series of subzero metabolic assays to examine its physiological potential under conditions comparable to those in the basal ice. Cells of TG14 incorporated [^3^H]-leucine and [^3^H]-thymidine into acid-insoluble macromolecules when incubated at −15 °C in frozen melt water from either the clean or sediment-containing ice (Figure 5). During frozen incubation for 70 days in the clean ice melt water, TG14 incorporated an average of 1.5 ± 0.2 × 10^4^ and 4.0 ± 0.2 × 10^3^ molecules CFU^−1^ of [^3^H]-leucine and [^3^H]-thymidine, respectively. TG14 cells frozen in melt water from the sediment containing ice incorporated an average of 8.1 ± 1.1 × 10^3^ and 1.6 ± 0.3 × 10^3^ molecules CFU^−1^ of [^3^H]-leucine and [^3^H]-thymidine, respectively. For the clean ice samples, incorporation of [^3^H]-leucine and [^3^H]-thymidine was continuous and hyperbolic (R^2^ > 0.94; *p* < 0.0001) over the entire time-course. For the banded dispersed ice melt water, incorporation of [^3^H]-leucine was initially rapid, but high variability in the data resulted in a poor fit of the regression model (α = 0.05); however, [^3^H]-thymidine incorporation appeared to be partially hyperbolic (R^2^ = 0.41; *p* = 0.0455; Figure 5). The maximum rates of [^3^H]-leucine and [^3^H]-thymidine incorporation in samples of the clean ice occurred during the first 90 h at 1.5 ± 0.2 × 10^3^ molecules CFU^−1^ day^−1^ and 2.3 ± 0.9 × 10^2^ molecules CFU^−1^ day^−1^, respectively. For the banded dispersed ice meltwater incubation, the maximum rates of [^3^H]-leucine and [^3^H]-thymidine incorporation also occurred in the first 90 h at 2.5 ± 0.9 × 10^3^ molecules CFU^−1^ day^−1^ and 2.7 ± 1.0 × 10^2^ molecules cell^−1^ day^−1^, respectively. There was not a significant difference between the maximum rates of [^3^H]-leucine (*p* > 0.38) and [^3^H]-thymidine (*p* > 0.79) incorporation measured in the clean ice and banded dispersed ice. However, cells in the clean ice incorporated significantly more [^3^H]-leucine (*p* = 0.049) and [^3^H]-thymidine (*p* = 0.003) than incubations conducted in the banded dispersed ice over 70 days.

## 4. Discussion

Glaciers and ice sheets move under their own weight, entraining sediment particles and liquid water (*i.e.*, through regelation) from subglacial sources into the basal zone of the ice mass [12]. In some regions of East Antarctica, basal ice is estimated to constitute up to 50% of the total ice sheet thickness [45], and a globally significant pool of organic carbon (21,000 Pg) is hypothesized to be buried beneath the Antarctic Ice Sheet [46]. Although the long-term fate of this material is difficult to predict, geomicrobiological investigations of basal ice environments have produced strong lines of evidence that suggest microorganisms may be an important and active component of these systems [13,14,15,16,17,28]. Understanding the extent and nature of microbial processes in basal ice environments is critical for understanding biogeochemical cycling in the cryosphere and relevant to discussions regarding microbial life in extraterrestrial frozen environments, e.g., Mars [47].

Analysis of banded dispersed basal horizons from Taylor Glacier revealed a simultaneous increase of CO_2_ (up to 325,000 ppmv) with a decrease of O_2_ (as low as 4% of total gas volume) with respect to atmospheric concentrations (*r* (6) = −0.92, *p* < 0.005; Figure 2) [30,31,48]. These depths were associated with increased cell concentrations (Figure 2A), suggesting that the entrapped CO_2_ and O_2_ concentrations may have resulted from the aerobic respiration of organic matter. Indeed, the phylotypes identified in this study were related to predominantly aerobic microorganisms, and only two genera, *Bacillus* and *Paenibacillus*, are known to contain facultatively anaerobic species. These observations are similar to those in other basal ice environments, where similar gas anomalies have been reported. For instance, Souchez *et al*. [49] reported elevated CO**_2_** (up to 130,000 ppmv) and CH_4_ (up to 6,000 ppmv) concentrations in basal ice from the Greenland ice sheet. Likewise, Campen *et al*. [17] found excesses of CO_2_, N_2_O and CH_4_ (32-, 240- and eight-times higher than atmospheric values, respectively) in ice from the Sajama glacier (Bolivia) and argued they resulted from microbial activity in the ice.

Molecular analysis of 16S rRNA genes amplified from Taylor Glacier basal ice revealed a low diversity of phylotypes from two bacterial phyla: the Firmicutes and Gammaproteobacteria. Although microorganisms attached to sediment particles in the basal ice may have been underrepresented in the 2009 samples, due to the sediment extraction procedure used, OTU diversity in the 2007 and 2009 was similar (H’ = 1.81 and 1.78, respectively). The diversity of OTUs in the banded dispersed basal ice (H’ = ~1.8) was significantly lower than that reported for Arctic permafrost (H’ = 3.6; [50]) and similar to values found in Antarctic sea ice (H’ = 1.1; [51]). In general, the molecular data are consistent with the low species richness observed in basal ice from the Bench Glacier (Alaska), the John Evans Glacier (Nunavut, Canada), and Greenland basal ice [26,52,53]. However, the subglacial environments of these glaciers appear to be largely dominated by members of the Betaproteobacteria (constituting between 25 to 51% of their clone libraries). From a phylogenetic perspective, our results are more similar to studies of permanent ground ice and permafrost in the Canadian Arctic, which found that the microbial assemblages inhabiting these frozen substrates were largely dominated by members of the Firmicutes (64% and 59%, respectively; [54,55]).

The most abundant phylotypes in the debris-rich ice horizons were affiliated with the genus, *Paenisporosarcina* (Figure 4). Enrichment culturing efforts were successful in obtaining a variety of psychrotolerant isolates with 16S rRNA gene identities >97% identical to the *Paenisporosarcina-*related phylotypes, as was also the case in previous culturing efforts on the Taylor Glacier basal ice [56]. Whether detected using molecular or culture-dependent approaches, members of the *Paenisporosarcina* have been documented in basal ice from three tunnel locations (~0.5 km apart) along the north margin of the Taylor Glacier. Some members of the Firmicutes, including species of *Paenisporosarcina* (e.g., *Ps. macmurdoensis*, *Ps. indica* and *Ps. quisquiliarum*), have the ability to differentiate into an endospore that is highly resistant to variety of environmental stresses. As such, their abundance in the ice could be attributed to selection, whereupon cells existing as environmentally durable spores would be more likely to persist in the ice after entrainment. However, results from pasteurization experiments indicated that 96% of the CFU were sensitive to heating at 80 °C, suggesting that the vast majority of the isolates obtained did not originate from heat resistant spores. 

Members of the *Paenisporosarcina* have been documented in a variety of permanently cold environments, including permafrost [50,55], arctic saline springs [57], alpine glaciers [58] and the McMurdo Dry Valleys [59], implying this group may have specific adaptations to low temperature conditions. Due to the concentration of solutes into the premelt phase during freezing, microorganisms found in the unfrozen fraction of ice are subjected to considerable osmotic stress. For example, at −15 °C, the molarity of an ice vein environment is estimated to be approximately 4.5 M [2]. All of the *Paenisporosarcina*-related isolates from this study exhibited moderate halotolerance between 6% and 12% (w/v). These levels of halotolerance do not appear to be sufficient for the estimated salt concentrations occurring inside Taylor Glacier basal ice veins (I = 4.6 M; [2]); however, the cells may be able to sustain a low rate of maintenance-oriented metabolic activity exclusive of biomass production or grow at rates lower than those measurable after 80 days of incubation.

Experiments were conducted in an effort to examine bacterial metabolism under conditions that simulated those within the basal ice. To ensure minimal carry-over of nutrients from the marine media, the harvested cells were washed and suspended in Taylor Glacier melt water. Marine broth contains 1,800 μg L^−1^ PO_4_-P, and if it is assumed that as much as 10 μL of residual supernatant remained in the tube between washes, the final solution would contain 72 pg L^−1^ PO_4_-P; a value ~1,400-fold lower than the lowest concentration measured in Taylor Glacier clean ice [30]. *Paenisporosarcina* sp. TG14 incorporated at a maximum rate of approximately 1,500 to 2,500 molecules of [^3^H]-leucine and 20 to 30 molecules of [^3^H]-thymidine per day. Assuming an average protein length of 267 amino acids with 7.3% leucine content per protein [5], the rates observed could support the synthesis of approximately four proteins every hour and the replication of the *Paenisporosarcina* sp. TG14 genome (3.83 Mb) once every ~20 years. The annual N and P requirement to support this rate of DNA and protein synthesis in the TG14 cell population is estimated at approximately 2 ng N y^−1^ and 50 fg P y^−1^. In terms of carbon turnover, these metabolic rates are extremely slow (0.81 to 2.1 × 10^−7^ gC gC^−1^ h^−1^), but would be sufficient for a maintenance metabolism at −15 °C directed towards sustaining vital cellular processes (e.g. osmotic regulation, pH regulation, repair and/or turnover of macromolecules) [60]. 

From the melted debris-rich basal ice (230–235 cm depth), the highest measured concentrations of dissolved organic carbon, dissolved inorganic nitrogen and dissolved inorganic phosphorus were 63 mg L^−1^, 0.68 mg L^−1^ and 5.5 μg L^−1^ respectively. These concentrations are approximately 10^2^-fold higher than those measured in the debris-poor clean ice [30]. Nutrient concentrations in the banded dispersed ice melt water used in the −15 °C incubation experiments were not directly measured; however, by inference from other measurements as previously described [30], they would have been higher than in the clean ice meltwater. These higher nutrient concentrations in the banded dispersed ice melt water did not significantly increase the rate of macromolecular synthesis by *Paenisporosarcina* sp. TG14 at −15 °C, as compared with cells frozen in the clean ice (Figure 5). Further, more radiolabeled substrate was incorporated in the clean ice incubations over the entire time-course. There are several explanations for these data; first, the TG14 cells may have preferentially used substrates in the banded dispersed ice or endogenous sources of leucine or thymidine in the samples. Secondly, lower amounts of incorporation in the banded dispersed meltwater data could be due to scintillation interference from fine particulates in the clay size fraction. Thirdly, higher values of precursor incorporation may have been a result of differences in the [^3^H]-leucine and [^3^H] thymidine concentrations in the ice veins. Vein diameters, and, therefore, unfrozen water volume in ice formed from dilute solutions (*i.e.*, the clean ice) would be smaller than those in ice formed from more concentrated solutions (*i.e.*, the banded dispersed ice). The unfrozen water volume of the clean ice is significantly smaller than that of the banded dispersed ice based on bulk concentration differences (I = 0.1 and 160 mmol L^−1^, respectively; [30]). As a result, 20 nmol L^−1^ of [^3^H]-leucine or [^3^H]-thymidine would exist at a higher concentration at −15°C in the liquid vein network in the clean ice. In support of this, micro-Raman spectroscopy of ice veins in clean Greenland glacial ice estimate that SO_4_^2−^ and NO_3_^−^ ions are ~10^4^ more concentrated than they appear in the melted bulk phase [61]. Mathematical modeling of the Taylor Glacier basal ice chemistry predicts that at −15 °C, ice vein solutes are only ~35 times more concentrated than in the melted bulk phase [2]. It should be noted that these values are based on mathematical models of ideal solutions, assume thermodynamic equilibrium (*i.e.*, no supercooling), and do not account for heterogeneity across a volume of ice.

In summary, basal horizons of the Taylor Glacier contained elevated concentrations of microbial cells, which consisted largely of bacteria in the phylum, Firmicutes. Species from the genus, *Paenisporosarcina*, were a numerically abundant member of this assemblage and appear to have characteristics that would promote survival in ice (e.g., psychrotolerance, halotolerance, metabolic activity to temperatures as low as −33 °C [21] and a spore-based survival stage). Our data support the notion that basal ice environments are active microbial habitats. Moreover, the habitability of frozen environments may be more favorable than previously recognized, as very low concentrations of nutrients appear capable of supporting metabolism in the unfrozen liquid phase of ice. Given a suitable microbial inoculum, the basal ice environment is a potential environment for biogeochemical cycling beneath glaciers and ice sheets. Our results supporting the persistence of microbial metabolic function at frozen temperatures also suggest that frozen worlds, such as Mars, Europa or Enceladus, could harbor cryogenic habitats suitable for microbial life [62].
